# Common cytotoxic chemotherapeutics induce epithelial-mesenchymal transition (EMT) downstream of ER stress

**DOI:** 10.18632/oncotarget.15150

**Published:** 2017-02-07

**Authors:** Parag P. Shah, Tess V. Dupre, Leah J. Siskind, Levi J. Beverly

**Affiliations:** ^1^ James Graham Brown Cancer Center, University of Louisville, Louisville, KY 40202, USA; ^2^ Department of Pharmacology and Toxicology, University of Louisville School of Medicine, Louisville, KY 40202, USA; ^3^ Department of Medicine, Division of Hematology and Oncology, University of Louisville School of Medicine, Louisville, KY 40202, USA

**Keywords:** ER stress, chemotherapeutics, EMT, migration, invasion

## Abstract

Endoplasmic reticulum (ER) in eukaryotes is a main organelle involved in a wide variety of functions including calcium storage, lipid biosynthesis, protein folding and protein transport. Disruption of ER homeostasis leads to ER stress and activation of the unfolded protein response (UPR). We and others have previously found that ER stress induces EMT in different cellular systems. Induction of ER stress with chemical modulators of ER homeostasis was sufficient to activate an EMT-like state in all cellular systems tested. Here, we provide evidence for the first time demonstrating that ER stress induces EMT that is neither cancer cell specific nor cell-type specific. In addition, we observed that chemotherapeutic drugs commonly used to treat patients also activate ER stress that is concomitant with activation of an EMT-like state. Interestingly, we find that following removal of ER stress, partial EMT characteristics still persist indicating that ER stress induced EMT is a long-term effect. Induction of mesenchymal characteristics, following chemotherapeutics treatment may be involved in providing cancer stemness and invasiveness in the cellular system. Interestingly, we find that mice treated with cisplatin have elevated level of ER stress and EMT markers in multiple tissues including lung, liver and kidneys. Furthermore, increased ER stress, as demonstrated by increased Bip, Chop, PDI, Ero1α and IRE1, and EMT, as demonstrated by increased Vimentin and Snail, is a hallmark of primary lung adenocarcinoma samples from patients. These observations have potential clinical relevance because overexpression of ER stress and EMT markers might contribute to chemoresistance and poor survival of lung adenocarcinoma patients.

## INTRODUCTION

Endoplasmic reticulum (ER) is a main eukaryotic multifunctional organelle involved in calcium storage, lipid biosynthesis, and protein folding and protein transport [1]. Misfolded proteins in ER are transported to cytoplasm where they get degraded by the proteasome through an ER associated protein degradation pathway (ERAD). Disturbance in ER homeostasis through ERAD leads to ER stress. Cells respond to ER stress by activating unfolded protein response (UPR), which activates downstream UPR related key components including PERK, IRE1α and ATF6 involved in maintaining and/or restoring ER homeostasis [2]. Moreover, ER stress has been shown to activate both apoptosis and survival pathways [3, 4].

Epithelial-mesenchymal transition (EMT) was first characterized during embryonic development in which cell lose epithelial characteristics and gain mesenchymal properties [5]. During EMT, cells undergo a phenotypic change which has been characterized by increased cellular motility and invasive properties. Cells lose their cell-to-cell contacts and become more elongated and migratory. Loss of E-cadherin in epithelial cells and increased expression of mesenchymal markers, such as Vimentin are hallmarks of EMT [6]. Several transcription factors including Snail, Slug, Zeb1/2 and SMAD-interacting protein 1 have been shown to be involved in EMT [5]. Moreover, several growth factors including hepatocyte growth factor, transforming growth factor-β and FGF-2 have been reported to be associated with several changes in epithelial cells, including loss of epithelial markers, acquisition of mesenchymal markers, and increased levels of ECM component [7, 8].

Recent *in-vitro* studies show that ER stress is one of the factors responsible for inducing EMT [9, 10, 11]. Moreover, studies have also demonstrated that pulmonary ER stress is involved in the pathogenesis of bleomycin-induced lung fibrosis and EMT in lungs [9, 12]. In the present study, using lung adenocarcinoma cell lines we show that thapsigargin and other well-known ER homeostasis modifiers induce ER stress and EMT. Interestingly, the ability of ER stress to induce an EMT-like state was not dependent on the tissue of origin or malignant status of the cells. Investigation into potential clinical relevance of these findings led us to examine whether or not common cytotoxic chemotherapeutic drugs induce ER stress and EMT. Cells were treated with cisplatin, cytarabine, doxorubicin, gemcitabine, Vinorelbine, Etoposide and Pemetrexed activated ER stress and ultimately EMT. Furthermore, we find that chemotherapeutic drug treatment including Gemcitabine, Doxorubicin and Cytarabine also increase cell migration and invasion of lung adenocarcinoma cells. Consistent with our *in-vitro* findings, we also observed elevated levels of expression of ER stress and EMT markers in multiple tissues including lungs, liver and kidneys of mice treated with cisplatin. Importantly, we observed that ER stress and EMT markers are commonly expressed in primary and metastatic lung cancer samples, when compared to normal lung tissue. In summary, the present study has provided evidence that ER homeostasis is a critical suppressor of EMT. Data suggest that disruption of ER homeostasis caused by chemotherapeutics may induce therapeutic resistance, tumor progression and even metastasis.

## RESULTS

### ER stress induces EMT

**Figure 1:**
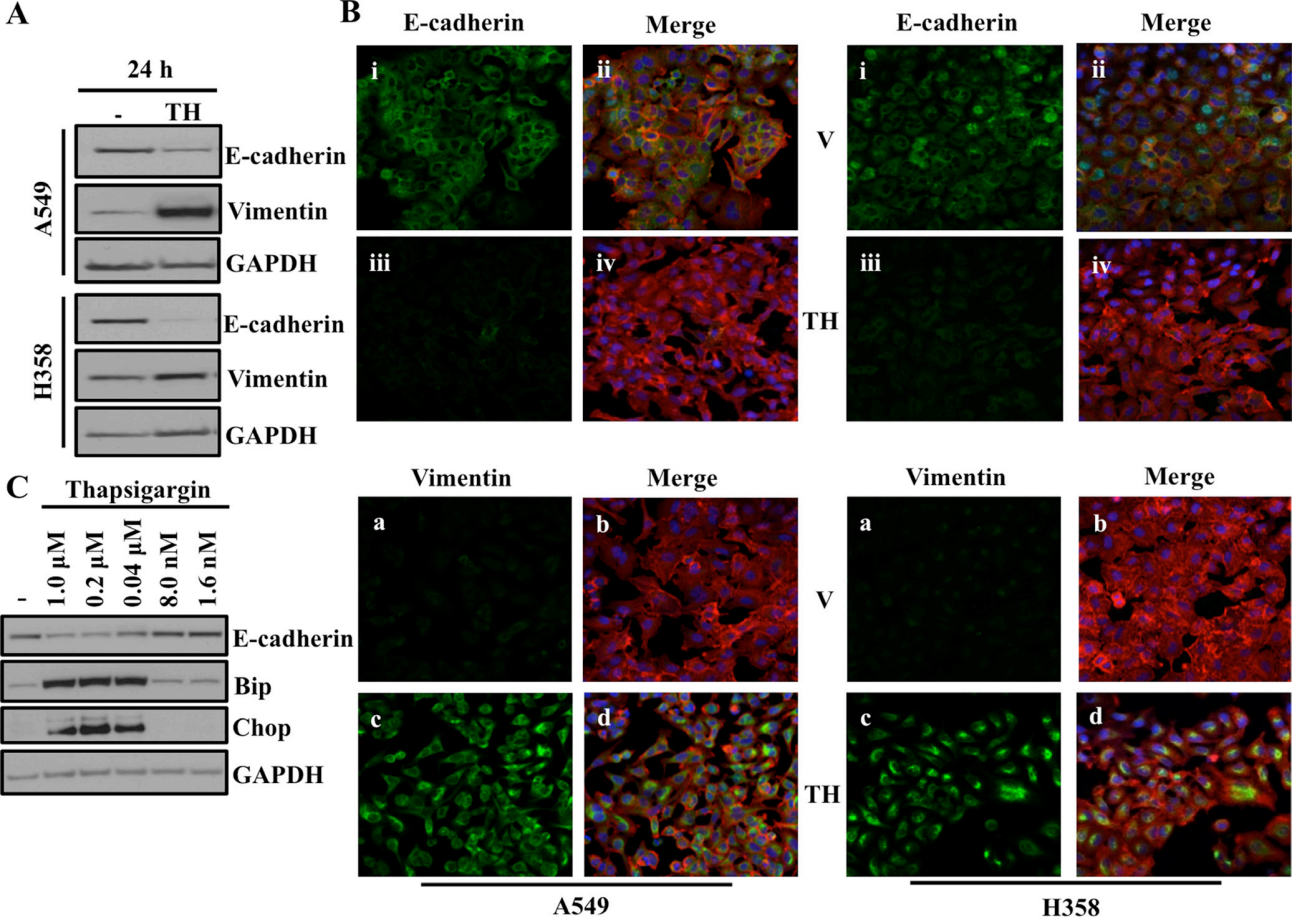
ER stress induces EMT (**A**) Western blot analysis of EMT markers in A549 and H358 cells. Cells were either treated with vehicle alone or with 0.5μM of thapsigargin for 30min and without thapsigargin in medium for 24hr. Cell lysates were prepared and western blot analysis of EMT markers Vimentin and E-cadherin was performed. (**B**) Fluorescence staining for E-cadherin and Vimentin in A549 and H358 cells. Cells were either treated with vehicle alone or with 0.5 μM of thapsigargin for 30 min and without thapsigargin in medium for 24 hr. After 24 hrs of treatment cells were trypsinized and plated on chamber slides and stained for EMT markers. i, and iii: E-cadherin was detected using Alexa Fluor 488 goat anti-rabbit IgG (green). ii and iv: overlay of respective E-cadherin and F-actin (Alexa Fluor 568 Phalloidin; red) staining with DAPI counter stain. a and c: Vimentin was detected using Alexa Fluor 488 goat anti-rabbit IgG (green). b and d: overlay of respective Vimentin and F-actin (Alexa Fluor 568 Phalloidin; red) staining with DAPI counter stain. (**C**) Determination of concentration of thapsigargin inducing ER stress response and EMT. A549 cells were treated with indicated concentration of thapsigargin for 24 hrs. A549 cell lysates were prepared and analyzed for ER stress and EMT markers.

We previously showed loss of AAA+ ATPase VCP is sufficient to cause endoplasmic reticulum (ER) stress, which ultimately induces EMT in lung adenocarcinoma cells [13]. Thus we wanted to determine if direct disruption of ER homeostasis via chemical disruption of ER function, could also induce EMT. To do this, we treated A549 and H358 cells with thapsigargin (TH), which is non-competitive inhibitor of the sarco/endoplasmic reticulum Ca2+ ATPase and is a well-known and well-studied ER stressor. Western blot analysis revealed significantly decreased expression of the epithelial marker E-cadherin and increased expression of the mesenchymal marker Vimentin following treatment with thapsigargin, indicating ER stress induced EMT in lung adenocarcinoma cell lines (Figure 1A). Thapsigargin-induced EMT in A549 and H358 cells was further confirmed by immunofluorescence staining for the EMT markers E-cadherin and Vimentin (Figure 1B). Again, E-cadherin staining was decreased and Vimentin staining increased following thapsigargin treatment of A549 and H358 (Figure 1B). Treatment with thapsigargin increased the expression of the ER stress markers Bip and CHOP at concentrations as low as 40 nM. Importantly, E-cadherin loss only occurred at concentrations of thapsigargin that induced ER stress as measured by activated Bip and Chop (Figure 1C). These data suggest that there is a close and parallel association between ER stress and EMT (Figure 1C) and demonstrate that ER stress can induce cells to adopt an EMT-like phenotype.

### ER stress-induced EMT is neither cancer nor cell type specific

**Figure 2:**
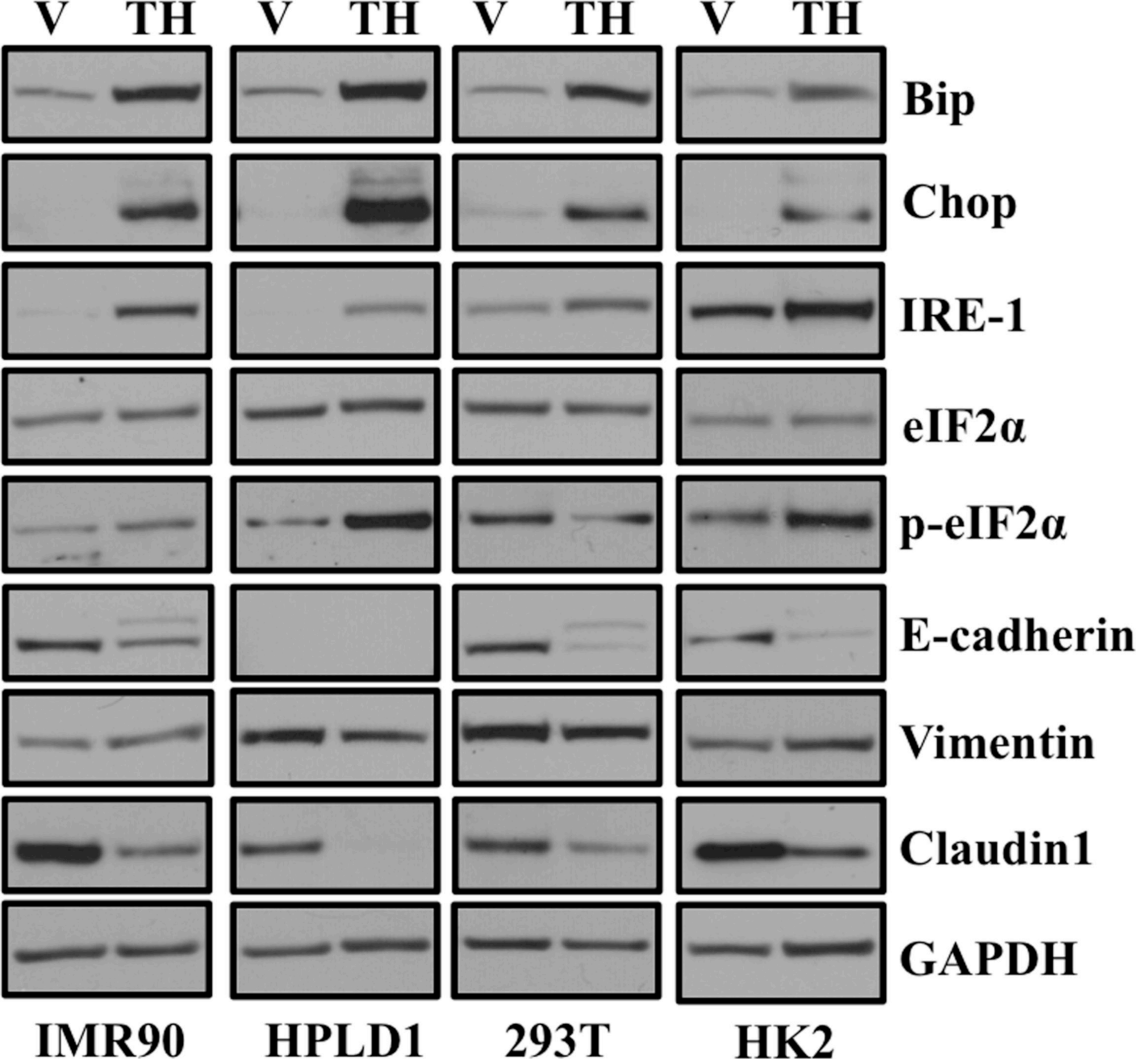
ER stress-induced EMT is neither cancer cell specific nor cell-type specific Cells were treated either with vehicle alone or with 0.5 μM of thapsigargin for 30 min and without thapsigargin in medium for 24 hr. Cell lysates were prepared and western blot analysis of ER stress response and EMT markers was performed. Western blot analysis of ER stress response and EMT markers in non-transformed human lung fibroblasts (IMR90), non-transformed human lung epithelial cells (HPLD1) and non-cancerous human kidney epithelial cells (293T) and an immortalized proximal tubule epithelial cell line from normal adult human kidney (HK2).

Previous studies have reported ER stress induced EMT in context of cancer [9, 13], however, it is unclear whether ER stress induced EMT is specific to cancer cells or cell types. Treatment of human lung fibroblast (IMR-90), immortalized human peripheral airway cells (HPLD-1), embryonic kidney (293T) cell lines and immortalized human kidney epithelial cells (HK2) resulted in a significant increase in expression of ER stress markers including Chop, Bip, and IRE1 (Figure 2). Furthermore, treatment with thapsigargin also induced an EMT-like status by decreasing expression of epithelial markers including E-cadherin and Claudin1 (Figure 2). However, we did not observe increase expression of mesenchymal marker Vimentin in HPLD1 and 293T cells. These observations suggest that ER stress induced EMT is a general phenomenon and is neither cancer specific nor cell-type specific.

### Additional ER stress inducing drugs causes an EMT-like phenotype; EMT is ER stress dependent event and EMT persists even after ER stress is resolved

**Figure 3:**
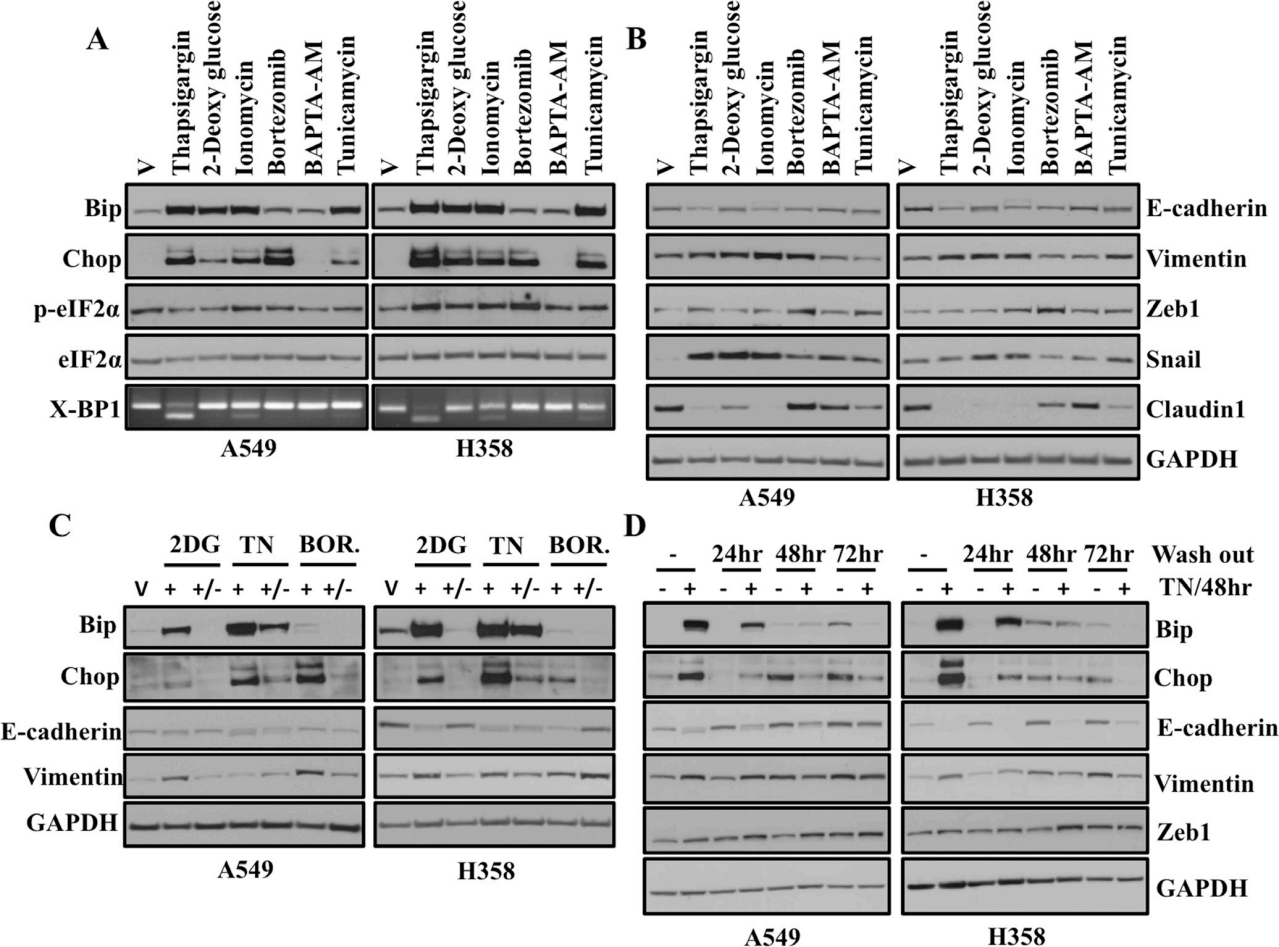
A variety of known ER stress inducing drugs cause an EMT-like phenotype that is reversible and EMT persists even after ER stress is resolved (**A**) Western blot analysis of ER stress response markers in A549 and H358 cells. Cells were treated with either vehicle or with indicated ER stress inducing drugs for 24 hr. Cell lysates were prepared and western blot analysis of ER stress response markers was performed. (**B**) Western blot analysis of EMT markers in A549 and H358 cells. Cells were treated either with vehicle or with indicated ER stress inducing drugs as mentioned above. Cell lysates were prepared and western blot analysis of EMT markers was performed. [Concentrations of ER stress inducing drugs used in the study. Thapsigargin: 0.5 μM, 2-Deoxy glucose: 40 mM, Ionomycin: 10 μM, Bortezomib: 20nM, BAPTA-AM: 50 μM, Tunicamycin: 1 μg/ml]. (**C**) A549 and H358 cells were either treated with vehicle or 2-Deoxy glucose (40 mM), TN (1μg/ml) and Bortezomib (20 nM) for 48 hrs, then drugs were washed out and cells were cultured for an additional 48 hrs in media without drugs. Cells were harvested at the indicated time points. Representative western blot analysis showing expression of proteins involved in ER stress and EMT. (**D**) Western blot analyses of ER stress and EMT markers in A549 and H358 cells. Cells were either treated with vehicle alone or with 1 μg/ml of tunicamycin for 48hr and without tunicamycin in medium for further 24 hr, 48 hr and 72 hr. Cell lysates were prepared and western blot analysis of ER stress and EMT markers were performed.

In order to ensure that the results we observed were not specific to thapsigargin, we extended our study to include many known chemical disruptors of ER homeostasis [2]. Confirming previously published results, we observed dramatic activation of ER stress following treatment of A549 and H358 cells with 2-deoxyglucose, ionomycin, bortezomib and tunicamycin (Figure 3A). Unfortunately, we did not observe increase in expression of ER stress markers with BAPTA-AM with the concentration used in our experimental condition. Activation of IRE1 results in splicing of mRNA encoding XBP-1 to produce a bZIP-family transcription factor which binds to promoters of ER chaperones [13]. In our study, we only observed splicing of mRNA encoding XBP-1 following treatment with known ER stress inducers including thapsigargin, ionomycin and tunicamycin in lung adenocarcinoma cell lines (Figure 3A). As we have suggested that ER stress induces EMT, we were interested to see whether these ER stress-inducing drugs also cause EMT. As expected, we observed increased expression of mesenchymal markers Vimentin, Snail and Zeb1 and decreased expression of epithelial markers including E-cadherin and Claudin1 following treatment of lung adenocarcinoma cell lines with drugs (Figure 3B). These results suggest that EMT induction is a common phenomenon downstream of ER stress. As we observed, EMT occurs only when ER stress is detected, we were interested to see what happens when ER stress is removed. For this, we treated A549 and H358 cells with either vehicle alone or with 2DG, TN and Bortezomib for 48 hrs and then the drug was washed out and cells were incubated for another 48 hrs without drugs in medium. Interestingly, we observed that EMT persists as long as ER stress is present and once ER stress is removed, EMT appeared to be reduced, but persisted suggesting that in this context EMT is an ER stress dependent phenomena (Figure 3C). Next, we were interested to see, how long an EMT-like state persists when ER stress is resolved. For that we treated A549 and H358 cells either vehicle alone or Tunicamycin for 48 hrs and then the drug was washed out and cells were incubated for another 24 hrs, 48 hrs and 72 hrs without drugs in medium. Interestingly, we observed EMT is ER stress downstream event and persists for even when ER stress is resolved (Figure 3D).

### Chemotherapeutic drugs activate markers of ER-stress and an EMT-like state

**Figure 4:**
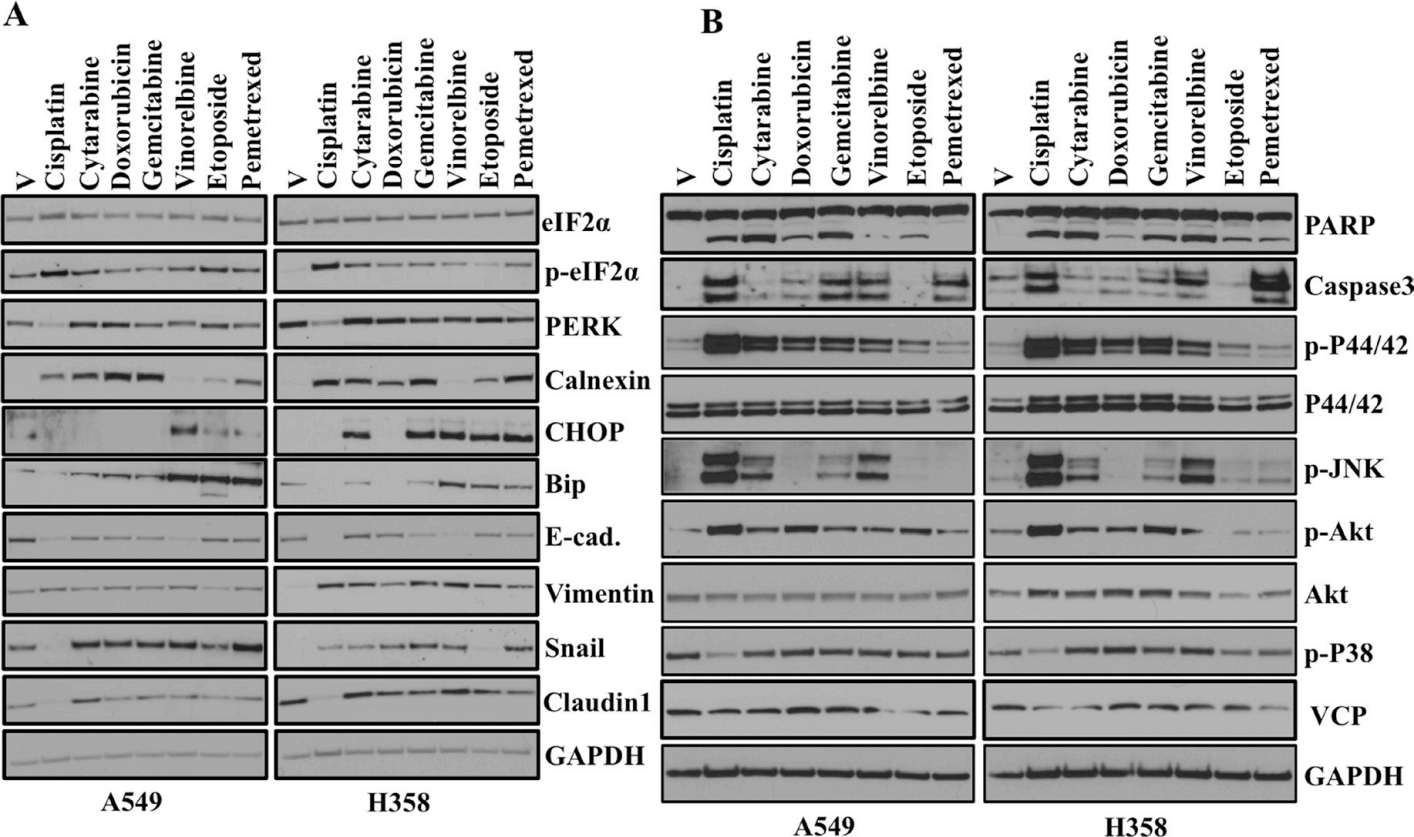
Chemotherapeutic drugs activate ER-stress, EMT and various signaling pathways (**A**) Western blot analyses of ER stress response and EMT markers in A549 and H358 cells. Cells were treated either with vehicle or with indicated chemotherapeutic drugs for 48hr. Cell lysates were prepared and western blot analysis of ER stress response markers was performed. [Concentrations of chemotherapeutic drugs used in the study. Cisplatin: 15 μM, Cytarabine: 2 μM, Doxorubicin: 1 μM, Gemcitabine: 2 μM, Vinorelbine: 0.05 μM, Etoposide: 2 μM, Pemetrexed: 0.2 μM]. (**B**) Western blot analysis of proteins involved in multiple signaling pathways in A549 and H358 cells. Cells were treated either with vehicle alone or with different concentration of chemotherapeutic drugs as indicated in A for 48 hr. Cell lysates were prepared and western blot analysis of ER stress response markers was performed.

The common mechanism involved in chemotherapy-induced apoptosis is generally DNA damage or cellular stress. Moreover, chemotherapeutic treatments have been shown to mediate cell death by activating key elements of the cellular stress response and apoptosis program [14]. Interestingly, recent studies have also reported positive correlation between EMT and chemoresistance [15, 16]. In the present study, we determine whether treatment with chemotherapeutic drugs activate ER stress and subsequent EMT induction. We treated A549 and H358 cells with chemotherapeutic drugs and assessed ER stress and EMT markers by western blot (Figure 4A). We observed a variable ER stress response following chemotherapeutic drug treatment as determined by an increase in Bip, Chop, Calnexin and PDI (Figure 4A). We also observed an increase in phosphorylation of eukaryotic initiation factor-α (eIF2α) downstream of Ser/Thr kinase PERK following treatment with chemotherapeutic drugs (Figure 4A). Treatment of the lung adenocarcinoma cell lines A549 and H358 with known and well established chemotherapeutic drugs including Cytarabine, Doxorubicin, Gemcitarabine, Vinorelbine and Pemetrexed not only induced ER stress (Figure 4A), but also promoted EMT as revealed by increase in expression of mesenchymal markers including Vimentin and Snail and decrease in epithelial markers including E-cadherin (Figure 4A). These data support a model in which ER stress, regardless of the mechanism of induction, promotes EMT.

### Chemotherapeutic drugs induce multiple signaling pathways *in-vitro*

In the present study, we showed that treatment with chemotherapeutic drugs activate ER stress and subsequent EMT induction (Figure 4A). Next, we were interested to see whether treatment with common chemotherapeutic drugs also activates different signaling pathways that are involved in different cellular events including apoptosis, cell proliferation and migration. Interestingly, western blot analysis revealed activation of Akt, JNK and Erk following treatment of the lung adenocarcinoma cell lines A549 and H358 with known and well established chemotherapeutic drugs treatment (Figure 4B). Most of the common cancer chemotherapeutics effect tumor cell killing *in-vitro* and *in-vivo* through the mechanisms of apoptosis [17]. Cleavage of PARP (poly ADP-ribose) polymerase facilitates cellular disassembly and serves as a marker of cells undergoing apoptosis [18]. Interestingly, we also observed increased in cleaved PARP following treatment with chemotherapeutic drugs. These data indicate chemotherapeutic treatment promotes apoptosis, but at the same time tumors that are resistant to chemotherapy are unable to activate the apoptotic machinery and may therefore become more aggressive and migratory following EMT induction. We previously showed role of AAA+ ATPase VCP, in the maintenance of the ER homeostasis in lung adenocarcinoma cells [13]. So, we were interested to see whether chemotherapeutic drugs treatment alter VCP expression. Interestingly, we did not see any significant change in expression of VCP following chemotherapeutic drugs treatment (Figure 4B).

### Treatment with chemotherapeutic drugs and thapsigargin induces cell migration and cell invasion

**Figure 5:**
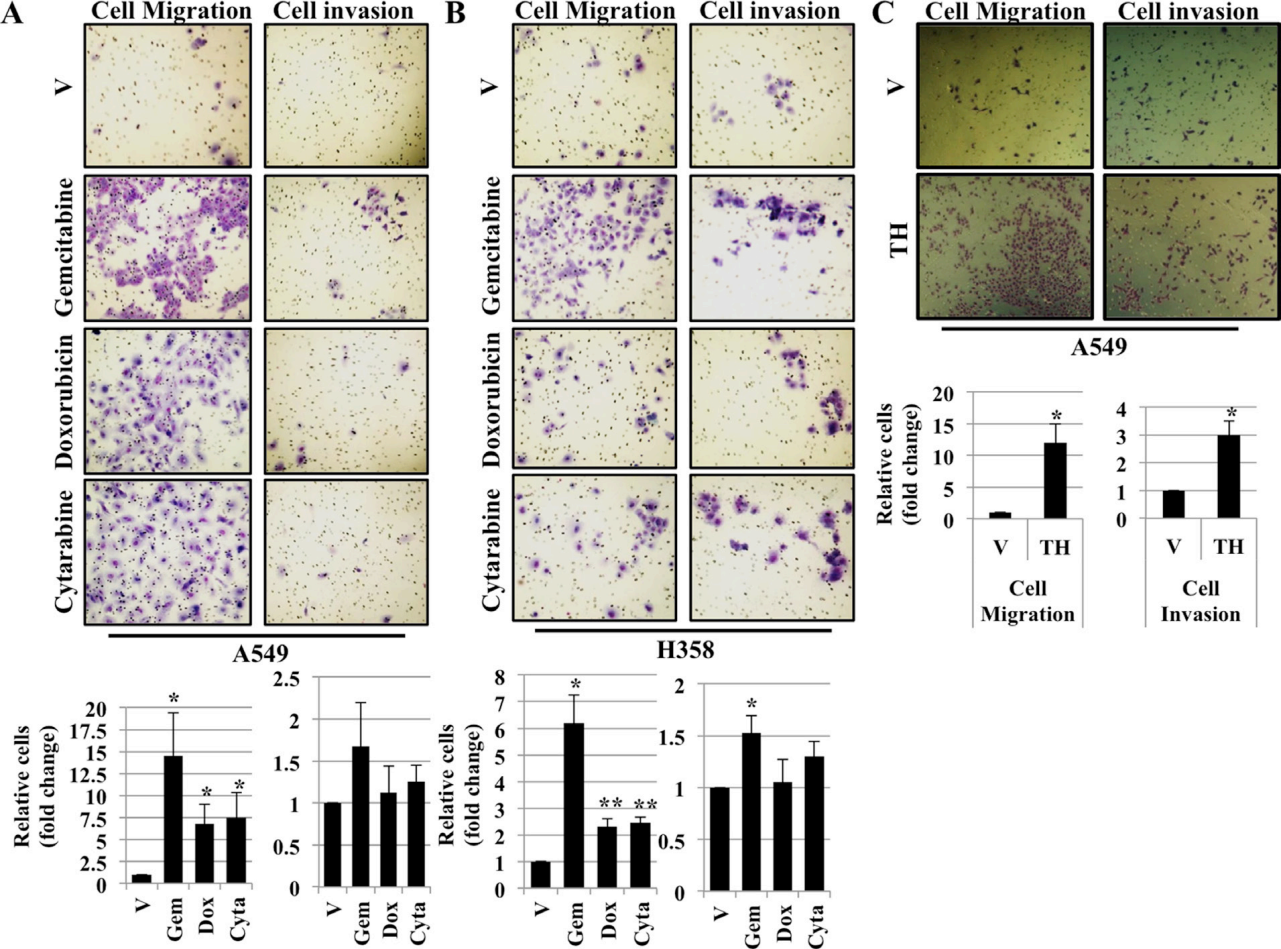
Treatment with chemotherapeutic drugs and thapsigargin induces cell migration and cell invasion Migration and Invasion assay in A549 (**A**) and H358 (**B**) cells. Cells were exposed to vehicle alone or Gemcitabine (2 μM), Doxorubicin (1 μM) or Cytarabine (2 μM) for 48 hrs. After 48 hrs cells were trypsinized, washed once with PBS, counted and seeded into Boyden chambers without (left) or with (right) matrigel. The lower chamber contained media with serum, whereas the upper chamber containing the cells was without serum. 48 hrs later cells on the underside of the membrane were fixed and stained. Quantification of relative number of cells migrated or invaded through matrigel (*P < 0.05) are depicted in lower panels. (**C**) Migration and Invasion assay in A549 cells. Cells were exposed to either vehicle alone or thapsigargin (0.5 μM) for 30 min and without thapsigargin in medium for 24 hr. After 24 hrs cells were trypsinized, prepared as described in (A) and (B) and seeded into Boyden chambers. 48 hrs later cells on the underside of the membrane were fixed and stained. Quantification of relative number of cells migrated or invaded through matrigel (*P < 0.05) are depicted in lower panels.

It has been shown that EMT is often associated with increased cell migration and invasion [19, 20]. During chemotherapy, many tumors do not respond completely to chemotherapy due to chemoresistance and tumor recurrence. The complete mechanism behind chemoresistance is poorly understood. In an attempt to determine how cells respond following ER stress, we performed cell migration and invasion assay. A549 and H358 cells were either treated with vehicle alone or with chemotherapeutic drugs including Gemcitabine, Doxorubicin and Cytarabine for 48 hrs. After 48 hrs, cells were trypsinized, washed and seeded in transwells and allow to grow. Interestingly, cells treated with chemotherapeutic drugs acquired more migratory and invasive phenotype as determined by the number of cells that migrated and invaded through matrigel compared with cells treated with vehicle alone, confirming that chemotherapeutic drug treatment resulted in increased cell migration and invasion (Figure 5A and 5B). Next, we were interested to see whether, treatment with thapsigargin leads to increase in cell migration and invasion. Interestingly, similarities in the result were observed following treatment with thapsigargin. A549 cells treated with thapsigargin acquired more migratory and invasive phenotype as determined by the number of cells that migrated and invaded through matrigel compared with cells treated with vehicle alone, confirming that thapsigargin (a disruptor of Ca+2 homeostasis, a SERCA inhibitor) treatment resulted in increased cell migration and invasion (Figure 5C).

### Chemotherapeutic drugs and thapsigargin treatment EMT and results in change in cell morphology

**Figure 6:**
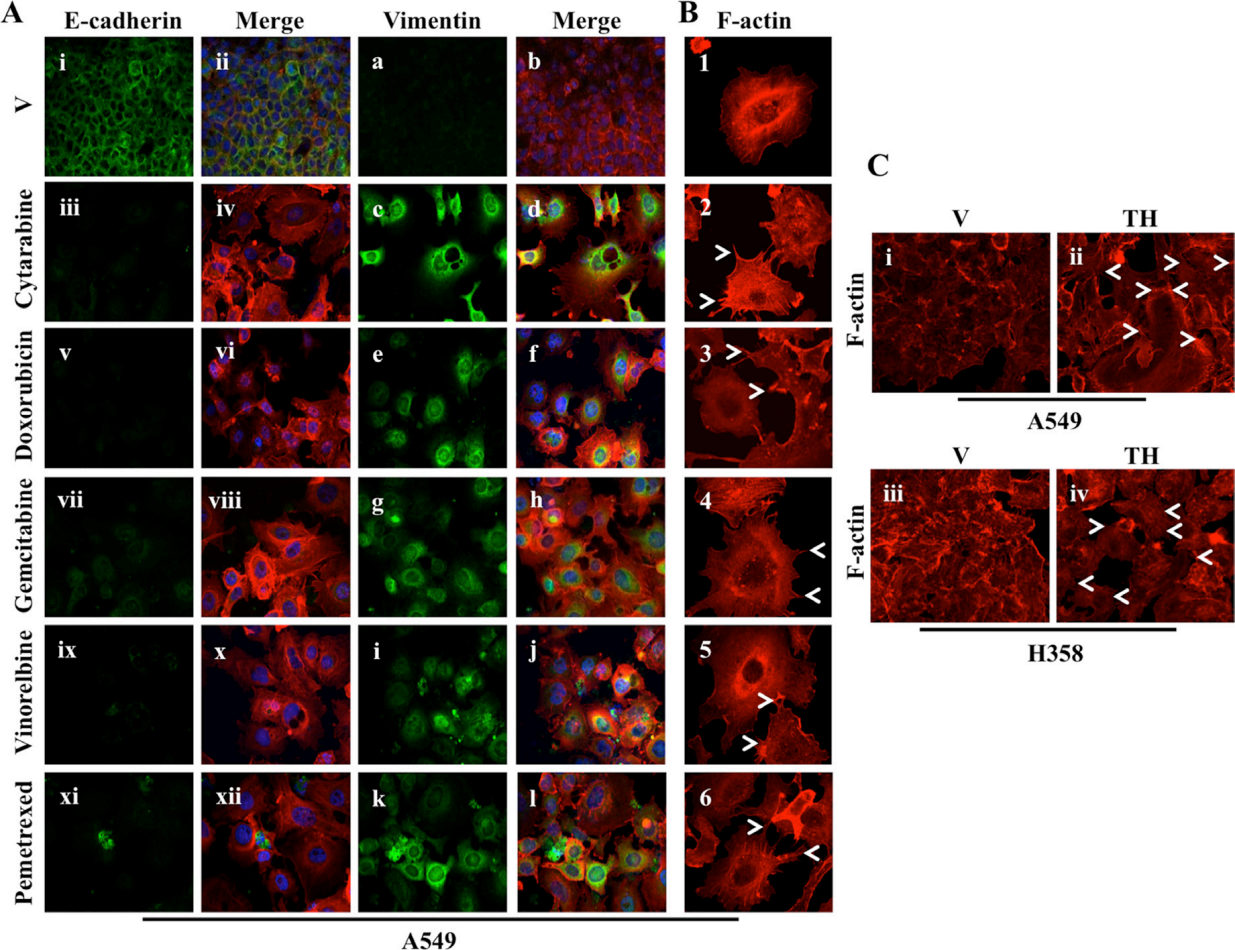
Chemotherapeutic drugs and thapsigargin treatment activate EMT (**A**) Fluorescence staining for E-cadherin and Vimentin in A549 cells. Cells were plated on chamber slides and either treated with vehicle alone or with known chemotherapeutic drugs with conc. indicated in Figure 4. After 48 hrs of treatment cells were fixed and stained for EMT markers. i, iii, v, vii, ix and xi: E-cadherin was detected using Alexa Fluor 488 goat anti-rabbit IgG (green). ii, iv, vi, viii, x and xii: overlay of respective E-cadherin and F-actin (Alexa Fluor 568 Phalloidin; red) staining with DAPI counter stain. a, c, e, g, i and k: Vimentin was detected using Alexa Fluor 488 goat anti-rabbit IgG (green). b, d, f, h, j and l: overlay of respective Vimentin and F-actin (Alexa Fluor 568 Phalloidin; red) staining with DAPI counter stain. (**B**) A549 cells were prepared as described in A and F-actin was detected with Alexa Fluor 568 Phalloidin (red). Re-organization of actin cytoskeleton through destruction and cellular protrusion formation is indicated by arrows. (**C**) Fluorescence staining for F-actin in A549 (i and ii) and H358 (iii and iv) cells. Cells were either treated with vehicle alone or with 0.5 μM of thapsigargin for 30 min and without thapsigargin in medium for 24 hr. After 24 hrs of treatment cells were trypsinized and plated on chamber slides and stained for F-actin (Alexa Fluor 568 Phalloidin; red). Re-organization of actin cytoskeleton through destruction and cellular protrusion formation is indicated by arrows.

We further confirmed that chemotherapeutic drug treatment activates EMT by decreased in expression of the epithelial marker E-cadherin and increased expression of the mesenchymal marker Vimentin by immunofluorescence staining in A549 cells (Figure 6A). Again, E-cadherin staining was decreased and Vimentin staining increased following treatment with different chemotherapeutics including Cytarabine, Doxorubicin, Gemcitabine, Vinorelbine and Pemetrexed in A549 cells compared to cells treated with vehicle alone (Figure 6A).

During EMT cells become more aggressive and acquire more invasive and migratory properties by changing cell morphology. We also observed reorganization of the cytoskeletal architecture, which results in change in cell morphology including cell elongation, membrane protrusions called lamellipodia and filopodia following treatment with chemotherapeutics (Figure 6B). Next, we were interested to see whether thapsigargin treatment leads to change in cell morphology. Interestingly, consistency in the results was observed with thapsigargin treatment further confirming that ER stress activates downstream EMT and results in cell morphology in lung adenocarcinoma cell lines (Figure 6C).

### Treatment of mice with cisplatin induces ER stress and EMT-like changes in multiple tissues

**Figure 7:**
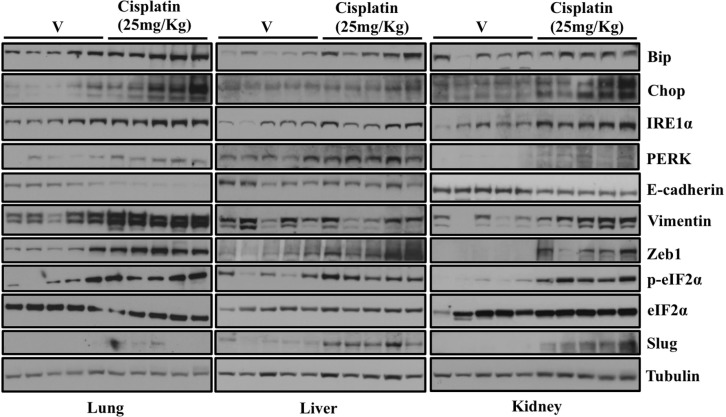
Treatment of mice with Cisplatin induces ER stress and EMT-like changes in multiple tissues C57BL/6 Mice were injected intra peritoneally (IP) with vehicle (5 mice) or Cisplatin (25mg/kg) (5 mice). After 48 hrs mice were euthanized and tissues including lungs, livers and kidney were collected and snap frozen in liquid nitrogen. Tissues were lysed in CEB lysis buffer. Representative western blot analysis showing expression of proteins involved in ER stress and EMT in the lungs and liver of mice either treated with vehicle or Cisplatin.

ER stress and its associated UPR and moreover EMT has been shown to be associated with cancer progression and metastasis [21, 22]. Since, we observed induction of ER stress and EMT following treatment with either known ER stress inducing agents or known chemotherapeutic drugs *in vitro*, we were interested to see whether there was a similar correlation *in vivo*. For that, we treated mice with well-known and commonly used chemotherapeutic drug, cisplatin. After 72 hrs mice were euthanized and tissues including lungs, livers and kidney were collected to analyze expression of proteins involved in ER stress and EMT by western blot (Figure 7). Studies have reported that EMT of tubular epithelium may be involved in cisplatin-induced renal fibrosis [23, 24]. Consistent with these findings, we observed that expression of ER stress markers including Bip, Chop, IRE1α, PERK and p-eIF2α were significantly elevated in lungs, livers and kidneys of mice treated with cisplatin compared to mice treated with vehicle alone (Figure 7). We also observed elevated expression level of mesenchymal markers including Zeb1 and Slug. More interestingly, we could see increase in the mesenchymal marker Vimentin and decreased expression of epithelial marker E-cadherin only in lungs and kidneys of mice treated with cisplatin compared with the mice treated with vehicle alone (Figure 7). We could not detect a difference in expression of E-cadherin and Vimentin in livers of mice treated with cisplatin. It has been shown that chemotherapy activates both apoptosis and survival pathways [25]. We also noticed activation of key proteins involved in both apoptosis and survival pathways *in-vitro* (Figure 4B). These data suggest that chemotherapeutic drug treatment lead to increased ER stress and EMT of normal cells *in vivo*.

### SRC kinase inhibitor PP2 blocks ER stress and EMT in lung adenocarcinoma cells

**Figure 8:**
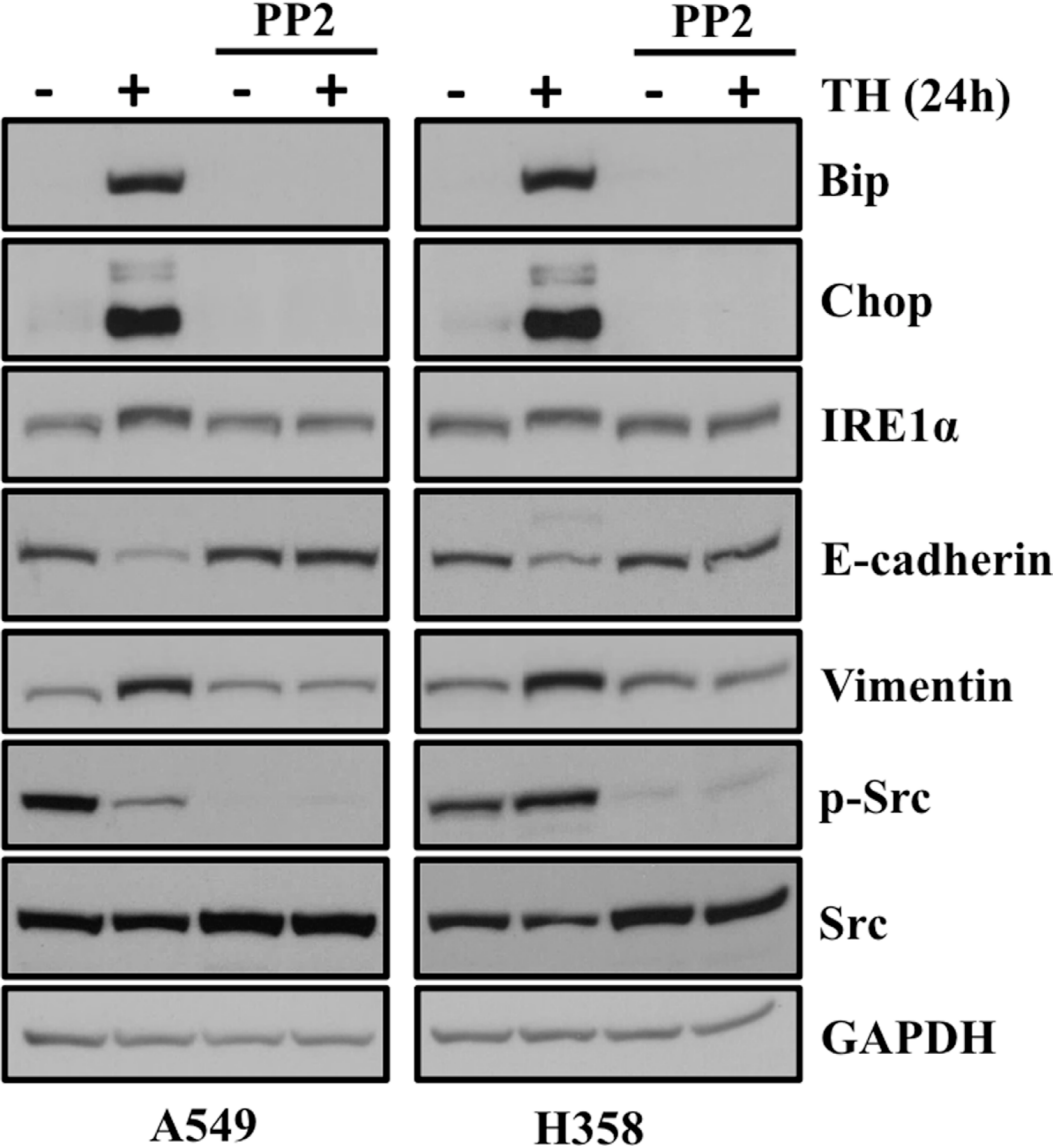
SRC kinase inhibitor PP2 blocks ER stress and EMT in lung adenocarcinoma cells treated with thapsigargin PP2 blocks ER stress and its associated EMT in lung adenocarcinoma cells. A549 and H358 cells were pretreated with PP2 (20 μM) and then cotreated with either vehicle alone or thapsigargin (0.5 μM) for another 24 hrs. Representative western blot analysis showing expression of proteins involved in ER stress and EMT.

We previously reported that either through siRNA mediated loss of AAA+ ATPase VCP or by its chemical disruption, cause ER stress and induces EMT in lung adenocarcinoma cells [13]. Furthermore we showed that well known Src kinase inhibitor PP2 blocks ER stress and subsequently EMT in lung adenocarcinoma cells. In the present study, we observed induction of EMT following ER stress either through chemotherapeutics or ER stress inducing agent. Next, we were interested to see whether treatment with SRC kinase inhibitor PP2 can abrogate or block ER stress and its downstream EMT in lung adenocarcinoma cells. Interestingly, we found that increased expression of ER stress markers including Bip and Chop following treatment with thapsigargin return to the basal level following treatment with PP2 (Figure 8). We also looked for expression EMT markers including E-cadherin and Vimentin. Interestingly, we observed that increased expression of mesenchymal marker Vimentin come back to normal level following PP2 treatment. Similarly, diminished expression of E-cadherin following thapsigargin treatment was restored following PP2 treatment further providing evidence that SRC kinase inhibitor, PP2 can be of potential use to block ER stress and EMT in lung adenocarcinoma cells (Figure 8).

### ER stress and EMT markers are co-expressed in primary patient lung tumor samples

**Figure 9:**
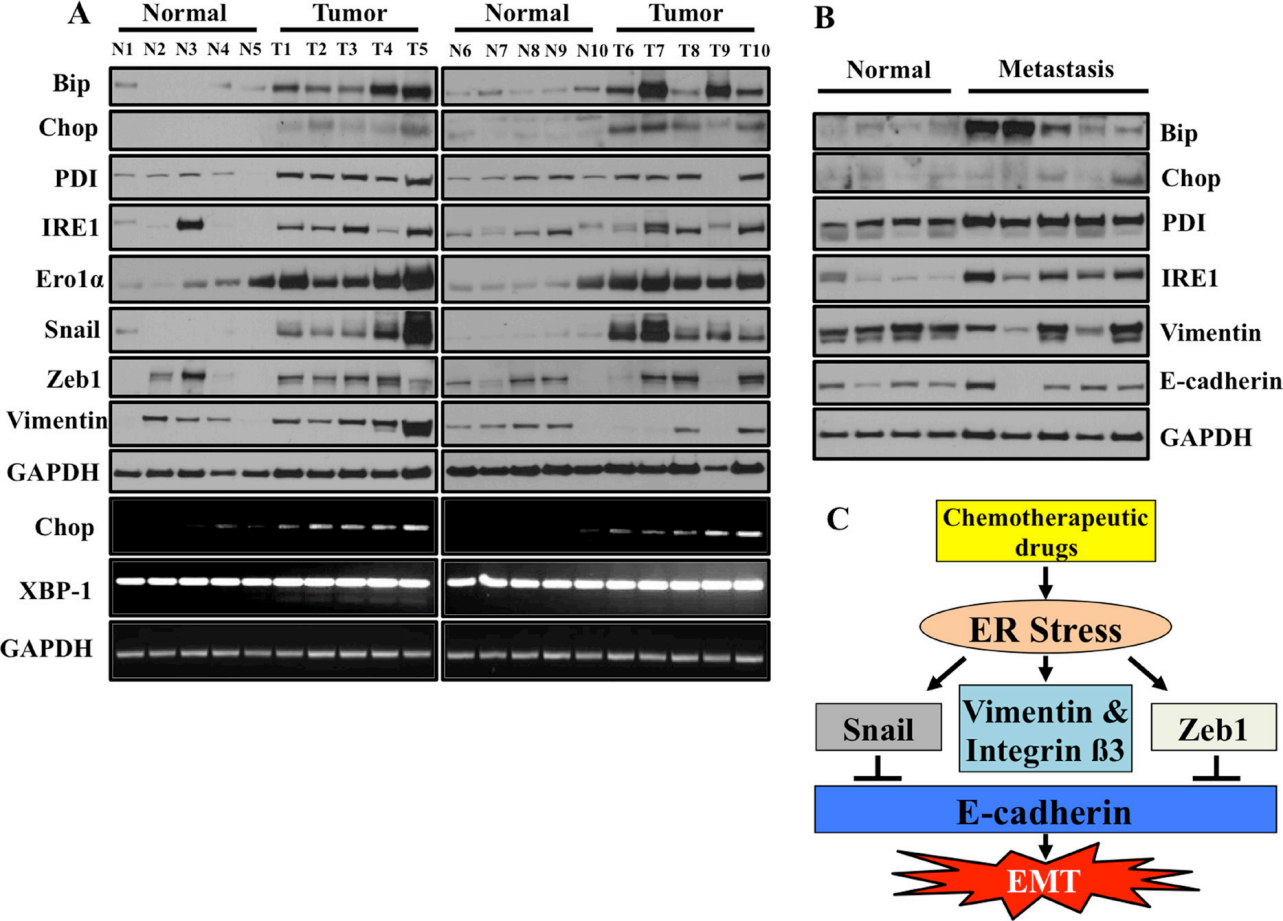
ER stress and EMT markers are co-expressed in primary lung tumors samples from patients (**A**) Western blot analysis of ER stress response markers and EMT markers. Snap frozen tumors from patient biopsies and normal adjacent tissues were lysed and western blotted for ER stress response markers and EMT markers. Samples represent matched tumor/normal pairs, such that N1 is the normal and T1 is the corresponding tumor from the same patient. (**B**) ER stress and EMT markers are overexpressed in primary lung metastasis compared to normal tissues. Frozen tumors from patients and normal lung tissues were lysed and western blotted for ER stress response markers and EMT markers. Samples do not represent paired tumor/normal samples. The normal samples are random specimens from our biorepository not analyzed in (A). (**C**) Schematic presentation of regulation of EMT by ER stress.

We have provided compelling evidence that ER stress drives EMT in human lung adenocarcinoma cells. In order to determine the clinical relevance of this *in vitro* data, we determined if ER stress and EMT markers are present in primary lung adenocarcinoma samples acquired from patient biopsies. We compared primary lung adenocarcinoma to normal adjacent lung from the same patient. Expression of the ER stress markers Bip, Chop, PDI, Ero1α and IRE1 was consistently elevated in nearly all lung tumor samples analyzed as compared to the adjacent normal (Figure 9A). Like ER stress markers, levels of mesenchymal markers including Snail, Zeb1 and Vimentin were also elevated in primary lung tumors as compared to adjacent normal tissues (Figure 9A). Interestingly, we also observed increased expression of CHOP and XBP-1 splicing at the mRNA level in primary lung adenocarcinoma compared to normal adjacent lung from the same patient (Figure 9A). Furthermore, ER stress and EMT markers were also present in metastatic lung cancer biopsies (Figure 9B). Unfortunately, the metastasis samples did not have matched biopsies from the primary site, but nonetheless when compared to a set of random normal lung tissues, dramatic increases in ER stress and EMT markers were observed (Figure 9B).

## DISCUSSION

ER in eukaryotes is an important organelle involved in calcium storage, lipid biosynthesis, protein folding and protein transport [1]. Environmental or physiological insults, as well as nutrient deprivation, disrupt the ER protein-folding environment and ultimately lead to accumulation of misfolded proteins within ER. This result in disruption in ER homeostasis and a condition termed ER stress. In order to compensate ER stress UPR gets activated through three major pathways including PERK, IRE1α and ATF6 [2].

Recently, interest has been developed elucidating how ER stress induced EMT could result in the pathogenesis of various diseases including cancer. A crucial step in beginning to fill the void in our knowledge is to elucidate how cells respond to cellular stress, such that under some circumstances the cell may activate a survival and EMT response, but if the stress is too great the stress may results in apoptosis [26]. In the present study we activate ER stress by treating lung adenocarcinoma cells with various ER stress inducers (Figures 1 and 2). We tried to determine if there was a concentration of TH at which it could activate low levels of ER stress, but not EMT. We were unable to find such a concentration. Interestingly, ER stress was either present or absent and any concentration of TH that induce ER stress, EMT markers were also activated. This study has demonstrated that ER stress induced EMT in lung adenocarcinoma cell lines, suggesting a potential mechanism whereby ER stress may contribute to cancer progression through regulating EMT.

So far, it was not clear whether this ER stress-induced EMT was cancer specific or epithelial cell type specific. In the present study, by utilizing multiple cell lines, we showed that ER stress-induced EMT is a general phenomenon that is not limited to cancer or specific epithelial cell types. Moreover, our study provided evidence that a variety of known ER stress-inducing drugs can activate EMT in lung adenocarcinoma cell lines. These include disruption of Ca+2 homeostasis (thapsigargin, a SERCA inhibitor; ionomycin a Ca^+2^ ionophore), inhibition of N-linked glycosylation (tunicamycin), nutrient deprivation (2-DG), inhibition of the proteasome (brotezomib), and cytotoxic stimuli (chemotherapeutics). Therefore, data presented in this manuscript indicate that regardless of the underlying cause for the ER stress, once it is initiated, ER stress promotes activation of EMT. Interestingly, we observed that ER stress activates an EMT-like phenotype as an initial response and this EMT-like state is maintained even if the ER stress is resolved, indicating that ER stress induced EMT is sustained effect. Detailed understanding of the processes and the mechanisms involved in ER stress induced EMT will be useful in future studies designed at deciphering the contribution that chronic ER stress plays in the progression of lung cancer.

Chemotherapeutic drugs generally act by promoting apoptosis by damaging DNA or inhibiting DNA synthesis leading to mitochondrial mediated death pathways. The ER is one of the most important compartments that play an important role in determining cellular sensitivity to cellular stress and apoptotic stimuli. Recently, it has been shown that chemotherapeutic drugs including cisplatin and gentamicin activate ER stress [27, 28]. In the present study we showed that treatment of various chemotherapeutic drugs, including cisplatin, activates ER stress by increasing the expression of chaperon proteins Bip, Calnexin, Chop, PDI and increased phosphorylation of eukaryotic initiation factor 2 (eIF2)α. Consistent with our findings, several studies have reported induction of ER stress markers, including grp78, are associated with anti-apoptotic function and resistance to anticancer drugs in non-small cell lung carcinomas [29, 30, 31].

Recent data demonstrate that EMT is involved in cancer progression. Further, metastatic disease is often refractory to chemotherapeutic treatments [32]. EMT is associated with the overexpression of several transcriptional factors such as Twist, Snail, Slug and Zeb1. Moreover, expressions of these transcriptional factors are known to be associated with shorter survival of the patients with lung adenocarcinoma [33]. In the present study, we have provided evidence that several chemotherapeutic drugs induce EMT-like phenotypic changes in lung adenocarcinoma cell lines. These changes included decreased expression of E-cadherin and increased expression of mesenchymal markers Vimentin and Snail. EMT is a major contributor towards metastasis and increased resistance to chemotherapeutics. Moreover, we show that following treatment of cells with chemotherapeutic drugs cells become more aggressive and acquire more invasive and migratory properties. We also observed reorganization of the cytoskeletal architecture, which results in change in cell morphology including cell elongation, membrane protrusions called lamellipodia and filopodia following treatment with chemotherapeutics and ER stress inducing agent thapsigargin (Figure 6B and 6C) [34].

Consistent with our *in-vitro* findings, we observed elevated levels of markers of ER stress and EMT in lungs, liver and kidneys of mice treated with cisplatin compared to mice treated with vehicle alone. Recent advancement in gene expression profiling has provided insights regarding the prevalence and association of EMT in primary human carcinomas. Prat and coworkers in their study involving breast carcinoma patients have positively correlated low expression of E-cadherin with high level of expression of mesenchymal markers including Snail, Twist and Zeb1 [35]. Similarly, it has been shown that colorectal carcinoma patients have elevated expression of mesenchymal marker including Snail and Zeb1 [36, 37]. Additional studies revealed that upregulated expression of mesenchymal marker including Snail and Slug are associated with chemoresistance in ovarian cancer cells [16] and furthermore silencing the expression of Snail or Twist can restore the sensitivity of A549 cells to cisplatin [38]. Consistent with these findings, we showed that ER stress and EMT markers are overexpressed in metastasis and primary lung tumor samples from patients, when compared to normal lung tissue. Although the sample size utilized in the present study was not extensive, we feel that they are representative of the disease. Further studies with increased sample size and more in depth clinical staging and outcome data will be helpful in assessing ER stress and EMT markers as biomarkers for assessing the overall prognosis and stage of patients with lung adenocarcinoma.

## MATERIALS AND METHODS

### Cell culture and protein analysis

Human lung adenocarcinoma cell lines A549, H358 and human normal lung fibroblast cells (IMR90; ATCC^®^ CCL186TM) were purchased from American Type Culture Collection (ATCC, Rockville, MD, USA) and cultured in RPMI medium supplemented with 10% fetal bovine serum (Invitrogen, Carlsbad, CA, USA) and 1% antibiotic/antimycotic (Sigma, St Louis, MO, USA). The human immortalized small airway epithelial cell line (HPLD-1) was cultured as described previously [39]. Human proximal tubule cells (HK2; ATCC^®^ CRL2190™) were cultured in keratinocyte serum free media (Invitrogen, Kit Catalog Number 17005042) supplemented with 0.05 mg/ml bovine pituitary extract (BPE) and 5 ng/ml of human recombinant epidermal growth factor (EGF). HK2 cells were utilized only at passages 2–6 so as to maintain their proximal tubule differentiated state. Human Embryonic Kidney cells (293T; ATCC^®^ CRL3216TM) were cultured in DMEM medium supplemented with 10% fetal bovine serum (Invitrogen, Carlsbad, CA, USA) and 1% antibiotic/antimycotic (Sigma, St Louis, MO, USA). The cell lines were routinely subcultured every 3–5 days. After experimental procedure cells were harvested in CEB lysis buffer # FNN0011 (Invitrogen, Life technologies, Grand Island, NY, 14072). Protein was quantitated by using Pierce's BCA Protein Assay Reagent Kit (# 23227) from Pierce Biotechnology, Rockford, IL, USA as per manufacturer's protocol.

### Western blot analysis and immunofluorescence staining

Western blots analysis and immunofluorescence staining was performed exactly as described previously [13]. Except, before performing immunofluorescence staining A549 and H358 cells were plated on chamber slides and treated either with vehicle alone or with TH (0.5 μM) for 30min and without thapsigargin in medium for 24 hr. Similarly, cells were also plated on chamber slides and treated with either vehicle alone or with different chemotherapeutic drugs including, Cytarabine (2 μM), Doxorubicin (1 μM), Gemcitabine (2 μM), Vinorelbine (0.05 μM) and Pemetrexed (0.2 μM). After 48 hrs of treatment either with vehicle alone or with chemotherapeutic drugs, cells were fixed with 4.0% paraformaldehyde in PBS for 30 min.

### Antibodies used for study

Bip #3183, CHOP #2895, eIF2α #9722 and p-eIF2α #9721, IRE1α #3294, PERK #5683, Ero1α #3264, JNK #9258, p-JNK 4668, PARP #9542, Calnexin #2679, PDI #3501, E-cadherin #3195, Vimentin #5741, Zeb1 #3396, Claudin1 #4933, Snail #3879, Slug #9585, Akt #9272, P-Akt #9271 (Cell Signaling Technologies Inc. Danvers, MA 01923); GAPDH #FL335 (Santa Cruz); α-Tubulin #T5168 (Sigma, St Louis, MO, USA). Alexa Fluor 488 goat anti-rabbit IgG #A11034 and Alexa Fluor 546 goat anti-rabbit IgG #A11010 (Molecular Probes, Invitrogen detection technologies, Eugene, OR. USA) and Alexa Fluor 568 Phalloidin #A12380 (Life technologies Eugene, OR. USA).

### Boyden chamber cell migration and cell invasion assay

Boyden Chamber cell migration and cell invasion assay was carried out as described previously [40]. Before performing assay, cells were exposed to vehicle alone or Gemcitabine (2 μM), Doxorubicin (1 μM) or Cytarabine (2 μM) for 48 hrs. After 72 hrs cells were trypsinized, washed once with PBS, resuspended in serum-free medium, and then seeded in Transwells (100,000 cells per Transwell). After 72 hrs, cells remaining inside the inserts were removed with cotton swabs and the cells that were migrated or invaded through the matrigel to the reverse side of the inserts were rinsed with PBS, fixed in 4% formaldehyde for 30 min at room temperature, and stained with Hema 3 stain. Similarly, in case of thapsigargin cells were exposed to either vehicle alone or thapsigargin (0.5 μM) for 30 min and without thapsigargin in medium for 24 hr. After 24 hrs cells were trypsinized, prepared as described previously and seeded into Boyden chambers. 48 hrs later cells on the underside of the membrane were fixed and stained.

### Animal experiments

Male C57BL/6J mice were purchased from Jackson Labs. The Animal Care and Use Committee of the University of Louisville approved all animal procedures. 8 week old male C57BL/6J mice were used for carrying out study. 5 mice were injected intraperitoneally (IP) either with vehicle (PBS) alone or with Cisplatin (25 mg/kg). After 72 hrs of treatment, mice were euthanized and tissues including lungs, kidneys and livers were collected and snap frozen in liquid nitrogen. Tissues were then lysed in CEB lysis buffer. Protein was estimated and equal amount of protein was used for studying expression of proteins involved in ER stress and EMT in the lungs and liver of mice either treated with vehicle alone or Cisplatin.

### Human primary tumor and adjacent normal lung tissue samples

Human primary tumor and adjacent normal lung tissue and metastatic tissue samples were obtained from tissue bio-repository facility of James Graham Brown Cancer Center, at University of Louisville. Local IRB committee of the University of Louisville approved the proposed human study. Tissues were lysed in CEB lysis buffer. Protein was estimated and equal amount of protein was used for studying expression of proteins involved in ER stress and EMT.

### RNA extraction, cDNA synthesis, and RT-PCR

For details regarding RT-PCR, Oligo sequences used for study and drug treatments see supplemental methods section.

## CONCLUSIONS

In conclusion, the present study demonstrates that ER stress caused by either chemical induction or chemotherapeutics is associated with EMT in a variety of cell lines, especially lung cancer cells (Figure 9C). These findings suggest an important and direct role for drug induced and chronic ER stress in lung cancer progression and metastasis. Furthermore, a complete understanding for how activation of ER stress induces EMT will be useful for the development of safer and more effective anticancer strategies. Future studies will be aimed at determining if we can block EMT activation downstream of ER stress, while maintaining the tumoricidal efficacy of chemotherapeutic agents.

## SUPPLEMENTARY MATERIALS FIGURES


