# Supplementary material for: Common cytotoxic chemotherapeutics induce epithelial-mesenchymal transition (EMT) downstream of ER stress
Source: Oncotarget. 2017 Feb 7;8(14):22625–39. doi: 10.18632/oncotarget.15150 (PMC5410250; doi:10.18632/oncotarget.15150)
Supplement: Supplementary file 1 [file oncotarget-08-22625-s001.pdf]

Common cytotoxic chemotherapeutics induce epithelial-mesenchymal transition (EMT) downstream of ER stress

Supplementary Materials

RNA extraction, cDNA synthesis, and RT-PCR

After 48 hrs of treatment either with vehicle alone or with TH (0.5 µM), 2-Deoxy glucose (40 mM), Ionomycin (10 µM), Bortezomib (20 nM), BAPTA-AM (50 µM) and TN (1 µg/ml), cells were harvested and total RNA was purified using TRI reagent (#9424) (Sigma-Aldrich, Inc. St. Louis, MO. USA), according to the manufacturer's protocol. The quality of the isolated RNA was assessed using the Bioanalyzer 2100 (Agilent Technologies, Inc., Wilmington, DE) and quantification was performed using a Nanodrop 1000 (Thermo Scientific, Wilmington, DE). cDNA synthesis and RT-PCR analysis was performed exactly as described previously [13].

Oligo sequences of XBP-1, CHOP and GAPDH used for study

Oligos for XBP-1, CHOP and GAPDH used for the study were ordered from Invitrogen, Life technologies, Grand Island, NY, 14072 USA. XBP-1 FP: CCTGGTTGCTGAAGAGGAGG, XBP-1 RP: CCATGGGGAGATGTTCTGGAG, CHOP FP: AGAA CCAGGAAACGGAAACAGA, CHOP RP: TCTCCTT CATGCGCTGCTTT, GAPDH FP: TGATGACATCA AGAAGGTGGT, GAPDH RP: TCCTTGGAGGCCAT GTGGGCC.

Drug treatments

A549 and H358 cells either treated with vehicle alone or with indicated concentrations of different drugs including thapsigargin (Sigma), tunicamycin (Sigma), 2-deoxy glucose (Cayman), Ionomycin (Sigma), proteasome inhibitor Bortezomib (Sigma) and BAPTA-AM (Sigma) for 24 hrs. In case of thapsigargin (TH) and Tunicamycin (TN), cells were either treated with vehicle or with indicated concentration of Thapsigargin and Tunicamycin (TN) for 30 min. After 30 min of treatment, TH and TN containing media was removed and fresh complete media was added to the cells, then cells were harvested after 24 hrs and total cell lysates were used for expression studies. Thapsigargin treatment in case of IMR90, HPLD1, 293T and HK2 cell lines was carried out in the similar fashion. Similarly, A549 and H358 cells either treated with vehicle alone or with indicated concentrations of different known and well established chemotherapeutic drugs including Cisplatin (Sigma), Cytarabine (Tocris bio), Doxorubicin (Cayman), Gemcitabine (Sigma), Vinorelbine (Sigma), Etoposide (Sigma) and Pemetrexed (Cayman) for 48 hrs. Cells were then harvested and total lysates were used for expression studies.
